# Supplementary material for: Tead1a Initiates Transcriptional Priming Through the TEAD1a/YAP‐Notch1‐Spi1/Cebpα Axis to Promote Neutrophil Fate
Source: Adv Sci (Weinh). 2025 Aug 28;12(41):e05441. doi: 10.1002/advs.202505441 (PMC12591127; doi:10.1002/advs.202505441)
Supplement: Supplementary file 4 — Supplementary Dataset [file ADVS-12-e05441-s004.zip › 3-6.zip!/3-6/02-6.cluster/cluster17/Enrich/ego_MF.pdf]

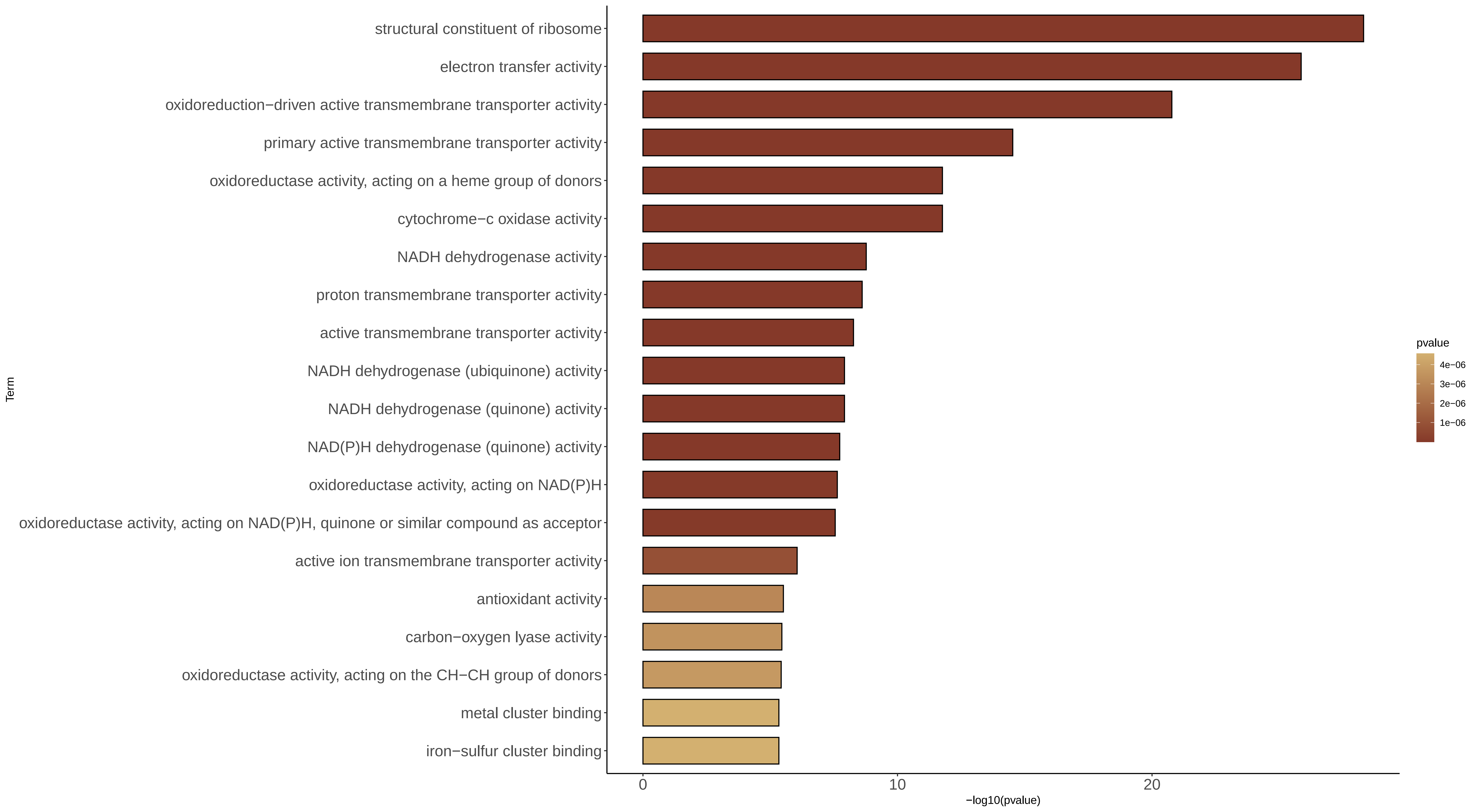
structural constituent of ribosome
electron transfer activity
oxidoreduction−driven active transmembrane transporter activity
primary active transmembrane transporter activity
oxidoreductase activity, acting on a heme group of donors
cytochrome−c oxidase activity
NADH dehydrogenase activity
proton transmembrane transporter activity
active transmembrane transporter activity
NADH dehydrogenase (ubiquinone) activity
NADH dehydrogenase (quinone) activity
NAD(P)H dehydrogenase (quinone) activity
oxidoreductase activity, acting on NAD(P)H
oxidoreductase activity, acting on NAD(P)H, quinone or similar compound as acceptor
active ion transmembrane transporter activity
antioxidant activity
carbon−oxygen lyase activity
oxidoreductase activity, acting on the CH−CH group of donors
metal cluster binding
iron−sulfur cluster binding
Term
0
10
20
−log10(pvalue)
pvalue
4e−06
3e−06
2e−06
1e−06
